# Heat-Related Illnesses Among U.S. Agricultural Workers from 2016 to 2024: Content Analysis of News Media Reports

**DOI:** 10.3390/ijerph23050549

**Published:** 2026-04-23

**Authors:** Christopher Benny, Jakob Hanschu, Roger G. Aby, Serap Gorucu, Bryan P. Weichelt

**Affiliations:** 1 Medical College of Wisconsin-Central Wisconsin, Wausau, WI 54401, USA; 2 National Farm Medicine Center, Marshfield Clinic Research Institute, Marshfield Clinic Sanford Health, Marshfield, WI 54449, USA; 3 Department of Agricultural and Biological Engineering, University of Florida, Gainesville, FL 32611, USA

**Keywords:** agriculture, farming, heat illness, outdoor workers, climate change, occupational safety and health, news media

## Abstract

**Highlights:**

**Public health relevance—How does this work relate to a public health issue?**

- Extreme heat is a major and rising cause of preventable illness and injury. Agricultural workers are disproportionately impacted due to the nature of their work requiring strenuous outdoor activity.
- Only a small number of media-reported agricultural heat-related injuries were identified in AgInjuryNews.org, suggesting that news media reports provide a limited and selective view of these events.

**Public health significance—Why is this work of significance to public health?**

- Between 2016 and 2024, only 14 media-covered agricultural heat-related injuries were located in the AgInjuryNews.org database and coverage largely prioritized fatalities. This can misrepresent the true burden and perceived risk of heat-related illness in agriculture.
- Even when news articles referenced Occupational Safety and Health Administration (OSHA) or laws/regulations, the references were brief and focused on investigation rather than prevention, missing a key opportunity to communicate actionable safety messaging, which can potentially prevent future incidents.

**Public health implications—What are the key implications or messages for practitioners, policy makers and/or researchers in public health?**

- The findings of this study highlight the clear need for agricultural health and safety professionals to collaborate with the media to improve the amount of prevention messaging included in heat-related illness coverage.
- The findings also highlight the limitations of exclusively relying on media reports to characterize heat-related injuries and the need for additional protections for these workers through public health policy initiatives.

**Abstract:**

In the U.S., extreme heat is the leading cause of weather-related fatalities. Farmers, ranchers and other outdoor workers who are exposed to the elements and engaged in strenuous physical activity are disproportionately impacted. This manuscript summarizes the number and severity of heat-related illnesses and injuries collected through the AgInjuryNews.org system, highlights their characteristics, provides recommendations for farmworkers and employers, and calls for future research. Heat-related illness cases from 2016–2024 were analyzed. Fourteen agricultural heat-related incidents covered by U.S. media were identified. Most incidents took place in June and July. A content analysis was conducted to identify news articles that included mention of prevention strategies, laws and regulations related to working conditions, or OSHA. Over half of the cases were from southern states. Eleven of the incidents involved male farmworkers, one involved a male farmer, and two involved first responders (gender unspecified). All of the farmer/farmworker incidents were single-victim fatalities. Seven articles mentioned prevention strategies, ten mentioned laws or regulations, and nine mentioned OSHA, often cursory. These findings suggest that media reports provide a limited and selective image of agricultural heat-related injuries, with coverage emphasizing fatalities and investigation information more often than prevention.

## 1. Introduction

Extreme heat is the leading cause of weather-related fatalities in the U.S. [1]. Unsurprisingly, outdoor workers who are exposed to the elements and engaged in strenuous physical activity are disproportionately impacted by extreme heat [2]. Agricultural workers have some of the highest rates of heat-related illnesses and fatalities [3,4]. Rising global temperatures, especially when combined with more extreme heatwaves, increase the risk of heat-related illnesses for U.S. agricultural workers [5,6,7,8,9,10,11,12]. Thus, heat-related illnesses are likely to be an important area of study and prevention for agricultural health and safety for decades to come. Appendix A (Table A1: Overview of Heat-Related Illnesses) includes a description of the various heat-related illnesses along with their pathophysiology and clinical manifestations.

Despite the increasing threat of extreme heat, comprehensive surveillance of heat-related illnesses in the agricultural sector remains limited. Current national datasets, such as the U.S. Bureau of Labor Statistics’ Survey of Occupational Injuries and Illnesses (SOII), are known to substantially undercount both fatal and non-fatal agricultural incidents [13,14,15,16]. Consequently, alternative data sources are needed to better characterize and understand these events.

This manuscript reviews and summarizes the number and severity of heat-related illnesses and injuries collected through the AgInjuryNews.org system between 2016 and 2024 and calls for future research in this field.

The key contributions of this work are as follows:

- It introduces a media-based approach for identifying and characterizing agricultural heat-related illnesses and injuries covered in U.S. news reports.
- It provides a descriptive summary of agricultural heat-related illness and injury cases captured in AgInjuryNews.org between 2016 and 2024.
- It highlights the selectivity and limitations of media-based case identification for agricultural heat-related illnesses.
- It demonstrates the potential value of news media analysis for identifying emerging trends, at-risk populations, and opportunities for targeted prevention and outreach.

It is important to note that this study does not aim to quantify the national incidence or prevalence of agricultural heat-related illnesses in the U.S. Rather, the study examines a subset of cases that were captured through a media-based surveillance approach. Hence, the number and geographic distribution of the cases analyzed are likely influenced by both true risk patterns and factors of newsworthiness that media organizations/professionals use to determine which events are reported on.

### 1.1. Risk Factors for Agricultural Heat-Related Illness

#### 1.1.1. Environmental Factors

Since the turn of the 20th century, the growing season for the contiguous 48 U.S. states has lengthened by an average of two weeks [17]. It is estimated that the average American agricultural worker currently experiences 21 dangerous working days due to heat exposure each year [18]. Intergovernmental Panel on Climate Change models estimate environmental temperatures will rise to 3.6 °F (2.0 °C) above the preindustrial baseline within the next 5–10 years, which would result in the average American agricultural worker experiencing an estimated 39 dangerous working days due to heat exposure [18].

#### 1.1.2. Occupational Factors

Occupational factors play a significant role in the development of heat-related illnesses among agricultural workers. Although mechanization has reduced the amount and intensity of manual labor required for production on many U.S. agricultural operations, physical exertion remains common, particularly in specialty crop sectors, where mechanized technologies have not yet replaced human labor [19]. Hats, gloves, rubber boots, and specialty gear like welding apparel or protective fruit-picking sleeves can all increase core body temperature, even as they protect against other hazards [20]. Much of this apparel and equipment exists in cooling or ventilated versions, but economic or ergonomic factors may impact whether those variants are used [20].

Access to shade, cooling stations, hydration stations, and scheduled breaks are some cooling measures that can be utilized to mitigate the risk of heat-related illness [16,21,22]. However, these measures are underutilized due to the costs associated with equipment, reduced productivity, and wages during breaks [18]. In some cases, even when these measures are available, practices such as piece-rate payment or the inefficient placement of cooling measures disincentivize workers from using them [23,24].

#### 1.1.3. Individual-Level Susceptibility Factors

##### Age-Related Risks

According to the U.S. Department of Agriculture Census of Agriculture, the average age of the U.S. farm producer population increased from 56.3 in 2012 to 58.1 in 2022 [25] Notably, the number of producers over the age of 65 increased by 12% from 2017 to 2022 [25]. While the average age for U.S.-born farmworkers remained roughly constant between 2006 and 2021, the average age of foreign-born farmworkers has increased by 7 years [25]. These trends among workers are concerning since people over the age of 65 are at a higher risk of heat-related illness [26].

Young children are also at a higher risk of developing heat-related illnesses [26]. The 2022 Childhood Agricultural Injuries (U.S.) Fact Sheet published by the National Children’s Center for Rural and Agricultural Health and Safety states that an estimated 893,000 youth lived on farms in 2014. Over half of youth living on farms also worked on the farm, and more than 265,600 non-resident youth were hired as farmworkers in 2014 [27,28].

##### Chronic Disease

Chronic medical conditions such as heart disease, poor blood circulation, and obesity increase a person’s risk of developing heat-related illnesses [26,29]. When individuals with chronic conditions participate in agricultural activities, they are often more prone to developing heat-related illnesses [30]. This also presents as a challenge when surveilling heat-related illnesses in agriculture, because heat exposure can exacerbate existing chronic conditions, and the medical visits linked to these exacerbations may not list the heat exposure leading to the visit [16].

##### Heat Acclimatization

Heat acclimatization or heat acclimation is a physiologic response to repeated elevations in skin and core body temperatures [31]. The temperature elevation can be due to increased ambient temperatures and/or physical activity [31]. The U.S. Department of Health and Human Services guidelines state that heat acclimatization requires a minimum of 2 h of heat exposure per day, and the best results are seen when gradually increasing the duration of physical activity in hot conditions over a period of 7 to 14 days while cooling off and rehydrating between shifts [32].

Heat acclimatization is generally maintained for a few days after the exposure to heat is halted but will begin to reduce after a week and is completely lost after a month of halting heat exposure [32]. Due to the seasonal nature of agricultural work in the U.S., workers are at an increased risk of heat-related illness when returning to work for the new growing season without heat acclimatization practices. California Occupational Safety and Health Administration investigations from 2005 showed that 46% of documented heat-related illness cases occurred on the employee’s first day on the job, and 80% of the cases occurred within the first 4 days of employment [33].

##### Knowledge, Training, and Behavioral Factors

Gaps in understanding among agricultural workers and employers on identification, prevention, and response to heat-related illnesses can contribute to increased incidence of these events [24,34,35,36]. Several studies show that agricultural workers have varying degrees of understanding of the importance of cooling treatments after heat exposure [24,34,35,36]. Beyond the pesticide safety training required under the Worker Protection Standard (WPS) by the U.S. Environmental Protection Agency, there are no nationwide regulations governing occupational health and safety training for agricultural workers [37]. In addition, variations in language and literacy levels amongst agricultural workers are a major barrier to adequate safety training [37].

##### Substance Use

The use of alcohol, caffeine, opioids, cocaine, amphetamines, methamphetamines, hallucinogens, benzodiazepines, and synthetic cathinone has been shown to increase the risk of developing heat-related illnesses [38,39,40]. Studies have shown that systemic factors contribute to prevalent alcohol and opioid use among farmers and farmworkers [41,42]. Agricultural workers are also likely to use caffeine to increase work efficiency and maintain alertness [24].

##### Migrant and Seasonal Farmworkers

According to the National Center for Farmworker Health, there are 2.9 million agricultural workers in the United States. Approximately 15% are migrant farmworkers and 85% are seasonal workers [43]. Migrant farmworkers are at an increased risk of heat-related illnesses due to language barriers, poor living conditions, low socio-economic status, lack of access to adequate personal protective equipment (PPE), geographical barriers to healthcare, unsafe working conditions, unjust treatment by employers, fear of deportation, racism, and cultural barriers, among many other factors [18,44,45,46]. These vulnerabilities may reduce the likelihood that incidents are reported and also influence how heat-related illness cases among migrant and seasonal farmworkers are captured in media-based surveillance systems.

### 1.2. Lack of Consistent Heat-Related Illness Protections

At present, there is no federal standard for occupational heat exposure. While certain states have implemented their own regulations, there are inconsistencies in the protections mandated by these regulations [36]. Five U.S. states have implemented regulations to safeguard workers from heat-related hazards: Minnesota, California, Oregon, Colorado, and Washington [16]. Minnesota was the first to establish a standard for indoor heat conditions, while California was the first to do so for outdoor conditions [16]. Washington, Oregon, and Colorado have regulations similar to California’s, mandating employers take measures to control and monitor heat-related injuries or illnesses [16]. In 2023, California proposed a new standard for indoor workplaces, and in 2024, Maryland introduced a proposed standard that covers both indoor and outdoor environments, which went into effect on 20 September 2024 [16,47]. Conversely, Florida and Texas have enacted laws restricting local governments to implement additional safety measures beyond those mandated at the state or federal level [36,48].

At the federal level, OSHA is currently proposing a standard titled “Heat Injury and Illness Prevention in Outdoor and Indoor Work Settings” to reduce heat-related risks and protect workers. The newly proposed standard will be applied to all employers in the general industry, construction, maritime, and agriculture sectors. It will require employers to create a comprehensive plan to evaluate and control heat hazards in their workplace. Additionally, the standard outlines specific measures that employers must take to protect employees from heat-related injuries and illnesses, ensuring a safer working environment for all [16]. This study does not evaluate the adequacy or effectiveness of existing protections; it only examines how such regulations and agencies are referenced in media coverage of heat-related agricultural injuries.

## 2. Materials and Methods

### 2.1. Dataset

AgInjuryNews.org is an online repository of U.S. and Canadian agricultural injuries, illnesses, and fatalities, primarily sourced from publicly available news media reports, including digital newspaper articles, television news websites, radio news websites, and other digital news outlets [15]. Relevant news articles are sources from keyword searches in media monitoring platforms such as Meltwater [49] and Google Alerts [50], as well as from submissions from users and collaborators, which are then verified by the AgInjuryNews.org team. The system has been collecting and coding these reports since its 2015 inception; current available data spans from 2016 through 2024, with new reports being added weekly. As mentioned earlier, U.S. Bureau of Labor Statistics occupational injuries and fatalities records present a significant undercount for the agricultural industry [13]. Thus, we analyzed the heat-related illness cases (2016–2024) in the AgInjuryNews.org dataset. It is important to note that AgInjuryNews.org also has limitations due to its content being dependent on the coverage of heat-related illnesses by news media and the system’s ability to locate these articles. However, it is an especially useful dataset for assessing the portrayal of agricultural heat-related illness cases in the U.S. media. Additionally, since the Bureau of Labor Statistics data is analyzed more frequently, this report draws from a different dataset to supplement understandings of the impact of heat-related illnesses and inform prevention strategies, particularly related to media messaging. Limitations and data collection and coding methods of the AgInjuryNews.org system are reported elsewhere [51,52,53]. A brief list of the limitations includes the selection bias introduced due to the publicly available media-dependent identification of incidents, reliance on coding/classification of incident details on the amount and clarity of information available in news reports, and inconsistencies/discrepancies in reports between sources. Since AgInjuryNews.org is dependent on publicly available injury reporting, the dataset for this study captures only a small, selective subset of agricultural heat-related injuries. The dataset identified for this study should not be interpreted as a representation of the true frequency or geographic distribution of agricultural heat-related injuries in the U.S. Rather, the dataset is best-suited for examining how these incidents are represented in publicly available news coverage.

### 2.2. Data Analysis

To achieve a more in-depth understanding of how agricultural heat-related illnesses are portrayed in the U.S. news media, the research team conducted a content analysis of the news articles associated with the identified agricultural heat-related illnesses. Content analysis is defined as “any technique for making an inference by objectively and systematically identifying specified characteristics of messages” [54]. The content analysis involved both deductive and inductive components. The deductive component sought to identify which articles included mention of prevention strategies, laws, and regulations related to working conditions or OSHA. The categorizations were determined a priori based on existing literature related to agricultural heat-related illnesses. The inductive component of the analysis sought to identify insights and patterns that emerged from the news reports. Microsoft Excel was used to complete the content analysis. Article information, including publication date, headline, and article text, was added to Excel with each row corresponding to an article. Characteristics corresponding to the incident reported in each article were also included in the rows: incident date, state, gender of those involved in the incident, and fatal/non-fatal outcome. For the deductive component of the analysis, presence/absence codes were assigned to each article according to whether it mentioned prevention strategies, laws or regulations, or OSHA. For the indicative component, theme or index codes were assigned to each article. This approach was chosen given the relative brevity of news reports compared to other forms of qualitative data as well as the exploratory and descriptive aims of the present project. Codes assigned to articles reflected the kinds of information reported in the article. These codes were refined based on multiple iterations of reading through the data as well as feedback from team members. Tabulated code frequencies as well as an article-by-code matrix informed the reporting of themes. All findings from both the inductive and deductive aspects of the content analysis were affirmed by multiple team members, including members not involved in the analysis.

## 3. Results

**Figure 1:**
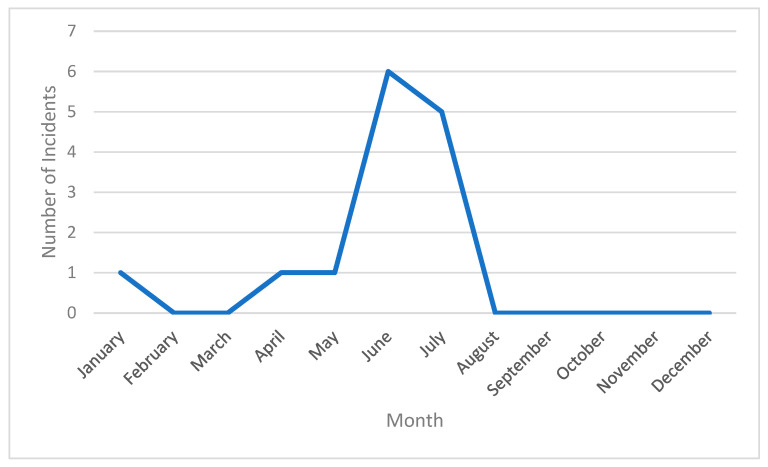
Monthly distribution of media-covered agricultural heat-related incidents identified in AgInjuryNews.org, 2016–2024.

**Figure 2:**
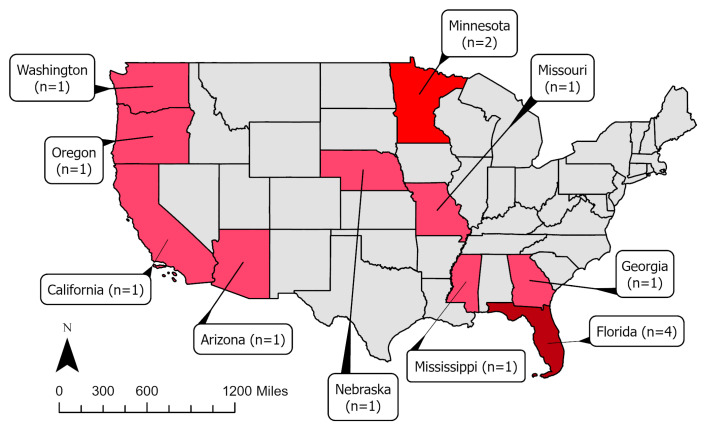
State distribution of media-covered agricultural heat-related incidents identified in AgInjuryNews.org, 2016–2024.

A total of 14 agricultural heat-related incidents were covered by U.S. media in the AgInjuryNews.org dataset. Table 1 provides summaries and characteristics of the incidents. Appendix B (Table A2: Narrative Summary of Agricultural Heat-Related Illnesses in AgInjuryNews.org, 2016–2024) provides a summary of the content included in the news reports corresponding to the 14 identified incidents. Of the 14 incidents, 12 took place from April through July, with most cases in June and July; Figure 1 provides a graphical representation of the temporal distribution of agricultural heat-related illnesses in AgInjuryNews.org from 2016 to 2024 by month. Unsurprisingly, over half of the cases came from states in the southern U.S.; Figure 2 provides a visual representation of the geographical distribution of agricultural heat-related illnesses in AgInjuryNews.org from 2016 to 2024 by state. Twelve of the incidents involved male farmworkers, one involved a male farmer, and two involved first responders (gender not specified). Age was provided for all but one of the cases involving farmworkers and farmers, and 42.7 was the median age. All of the incidents involving farmers and farmworkers were single-victim fatalities. The first responder incidents both involved firefighters performing a grain bin rescue. The fourteen incidents were covered by 19 U.S. news articles that were included in the analysis. Some incidents were covered by multiple articles, with later articles often providing updates to initial coverage.

Results of the deductive content analysis are shown in Table 2. Of the 19 articles covering 14 agricultural heat-related incidents, 8 included prevention strategies, 11 discussed laws or regulations (including critically), and 10 mentioned either state or federal OSHA agencies. Prevention strategies tended to be mentioned as general statements and were not linked explicitly to the cases described in the news reports. Interestingly, mentions of laws and regulations often lacked detail and were merely referenced as the reason for investigation. The reports that contained more detailed information about laws and regulations were often critical of the existing regulations for being inadequate. Similarly, OSHA was typically referenced as the body carrying out an investigation. In other words, little information about the content of existing OSHA standards or other regulations and laws was mentioned in the articles.

The inductive content analysis revealed that most of the reports sought to objectively describe the incident and did not assign blame or indicate the incident might have been preventable. Details about the identity of the victim and the task they were completing at the time of the incident were always included. The temperature or heat index at the time of the incident was generally provided as additional contextual information. Beyond those details, it was common for the news reports to focus on the ensuing investigation. Several reports reference increasing heat conditions and the need for more intensive regulations. However, criticism of (a lack of) existing regulations typically came from subject matter experts, such as public health researchers, that the media outlet contacted to provide a comment. One of the most troubling findings from the content analysis is that nearly a third of the news articles (6 of 19) report that, prior to the incident, the victim was either observed or had informed their manager or a fellow worker that they were feeling unwell. For example, here is a statement by an OSHA official in a *Miami Herald* article reporting on a farmworker fatality due to heat exposure: “The employee was observed exhibiting symptoms such as fatigue, weakness, and disorientation, [and was] struggling to keep pace with more experienced farmworkers.” [55].

## 4. Discussion

### 4.1. Media Representation and Data Gaps in Agricultural Heat-Related Illness

Several findings emerge from an examination of the U.S. media reports about agricultural heat-related illnesses covered in the AgInjuryNews.org dataset. First, there are relatively few agricultural heat-related illness cases reported in the articles captured by the AgInjuryNews.org database. We hypothesize that there are several reasons for this scant coverage within the database, including newsworthiness, abundance of non-fatal rather than fatal incidents, and the occurrence of these events on private property (in contrast with roadway incidents). The known issue of underreporting farmworker injuries and illnesses (especially migrant workers) is another likely contributor to the relatively scarce media coverage of agricultural heat-related illnesses within the AgInjuryNews.org database, since farmworkers are often the victims. When injury and illness events are not reported to regulatory agencies, it is plausible that they will not receive media coverage.

Second, within the news reports captured in the AgInjuryNews.org database, heat-related illnesses in agriculture were only covered if they involved a fatality. This pattern suggests that news coverage may emphasize the most severe events and may therefore provide a selective picture of agricultural heat-related illness to the public and policymakers. The only non-fatal heat-related illnesses covered in the news media were incidents involving first responders.

Third, at least according to our sample, agricultural heat-related illnesses disproportionately impact farmworkers, an already precarious group in the U.S. agricultural industry. This finding underscores the importance of continued attention to prevention among hired agricultural workers, specifically farmworkers who likely face structural vulnerabilities. The analysis revealed that less than half of the media articles about agricultural heat-related illnesses included prevention messages, even as several of them discussed how victims reported they were feeling unwell prior to their deaths. Thus, there is a major need and opportunity for agricultural health and safety practitioners to work with members of the media to improve prevention messaging, including information about signs that someone is experiencing the onset of a heat-related illness.

Generally, the articles analyzed portray agricultural heat-related illness cases as events to be *investigated* rather than as opportunities for *prevention* messaging. This pattern suggests that news coverage emphasizes the most severe events and therefore provides a selective picture of agricultural heat-related illness. We hypothesize that the newsworthiness of investigation-focused reporting likely plays a role in how heat-related illnesses are covered by journalists. However, the critical point is that an over-emphasis on investigation (i.e., “What happened?” or “Who is at fault?”)-absent prevention framing can distract from the fact that measures exist that might have prevented the event from happening at all. Relevant laws and regulations were almost never described in detail. More time (and text) was spent detailing investigative efforts than discussing OSHA or other standards and regulations and their application to the case or to agricultural operations generally. A crucial part of improving media coverage of agricultural heat-related illnesses involves clear communication about existing laws and regulations. Not only would such communication inform agricultural employers about their responsibilities, but it would also inform agricultural workers about their rights. Additionally, there may be value in complementary communication approaches to emphasize prevention while still leveraging the visibility of specific incidents. Lastly, the small number of cases identified in this media-based dataset reinforces that the exclusive use of news reports is insufficient for characterizing the full scope of agricultural heat-related illnesses. Future work should examine how media-based approaches can complement other data sources and how prevention-focused messaging can be more consistently incorporated into public reporting.

### 4.2. Recommendations for Farmworkers and Agricultural Employers

The Occupational Safety and Health Act recognizes heat as a preventable workplace hazard [16]. There are several studies, reports, and resources that provide guidance on how to effectively prevent heat-related illnesses [16,18,21,22,33,35,36,56,57,58,59,60,61]. For this article, we would like to highlight some recommendations to reduce the risk of developing heat-related illnesses when performing agricultural work. Appendix C (Table A3. Evidence-Based Strategies for Preventing Heat-Related Illnesses in Agricultural Work) includes the key recommendations the authors of this manuscript would like to highlight. This is by no means a comprehensive list, and additional resources should also be consulted. To effectively implement the strategies listed in Table A3, we strongly recommend the use of measurable indicators that can be used to evaluate the effectiveness of the interventions and make changes as needed. Some examples of measurable indicators include- creating systems to record incidence of heat-related symptoms, such as the ones mentioned in Table A1; collecting near-miss reporting frequencies; tracking emergency medical service activation timing; and internal compliance audits of hydration, rest, and shade availability. For the data to be useful, employers must have systems in place to enact and track practice/policy changes based on indicator data.

### 4.3. Automation in Agriculture and Its Implications for Heat-Related Illnesses

In the coming decades, more farmers will likely turn to automation, using technologies such as Artificial Intelligence (AI), autonomous machines, sensors, and other digital hardware, as well as software, to increase efficiency and precision, boost productivity, and meet rising food demands in a sustainable way that is less dependent on human labor. Autonomous machines such as driverless tractors, drones, and robotic harvesters would significantly reduce the number of farmworkers and the amount of time they are exposed to high-temperature environments by mechanizing physically demanding tasks such as planting and seeding, harvesting, weeding, pruning, sorting, and packing [62,63]. As a result, the incidence of heat-related illnesses among agricultural workers involved in these activities would be expected to decline [64,65].

However, automation cannot completely remove humans from farm operations, especially in rural settings where the lack of infrastructure and technical malfunctions may require workers to monitor or repair the autonomous machines under extreme temperatures. Furthermore, because autonomous machines often operate continuously, using such machines would eventually change farm operation schedules, leading to human maintenance or supervision needs during times when human labor would not typically be engaged [66]. Additionally, implementing autonomous systems may unintentionally result in labor segmentation with marginalized workers (i.e., low-wage laborers, migrant farmworkers, workers without access to advanced training or education) performing the more hazardous and physically demanding tasks that autonomous machines cannot handle, including tasks that require manual interventions outdoors, monitoring outdoor operations, and repairing equipment [67]. Yet another consideration is that some forms of automation, especially those utilizing AI, may contribute to rising global temperatures due to energy consumption or the resources required to produce them [68,69].

Beyond fully autonomous machinery, the future of agricultural automation is increasingly expected to involve human–robot collaborative systems, including wearable robotics, agricultural cobots, and semi-autonomous platforms that require continuous human interaction [66]. These technologies may have significant implications for the risk of heat-related illnesses. By reducing metabolic workload, physical exertion, and repetitive strain, wearable robots and cobots could lower core body temperature elevations and cardiovascular stress among workers exposed to hot environments [67,68]. Studies in industrial and military settings suggest that lower-energy-demand tasks mediated by robotic assistance are associated with reduced heat strain and fatigue, even under high ambient temperatures [67]. Translating these systems to agricultural settings could therefore offer protective benefits, particularly during harvesting and manual crop handling tasks that currently contribute substantially to heat-related morbidity.

However, the integration of human–robot systems may also introduce new risks. Wearable robotics and close-proximity robotic systems often increase thermal insulation, restrict airflow, or add weight, potentially exacerbating heat retention if not designed for hot environments [67]. Moreover, prolonged human–robot interaction may require sustained outdoor presence, limiting opportunities for rest or cooling if work pace increases due to perceived efficiency gains [68].

Overall, to maximize the potential of human–robot technologies to reduce heat-related illness, future implementation should prioritize heat-aware system design, including lightweight materials, passive or active cooling, ergonomic optimization, and integration with real-time physiological monitoring. Regulatory frameworks and occupational health policies should also explicitly address human–robot collaboration in hot environments, ensuring that productivity gains do not come at the expense of worker safety. As these systems become more widespread in agriculture, targeted research evaluating their net impact on heat exposure, physiological strain, and injury risk across diverse worker populations will be essential.

### 4.4. Limitations

AgInjuryNews.org data was used for the purposes of this report due to the reported undercount of illnesses and injuries included in the Bureau of Labor Statistics SOII for the agricultural industry. This undercount in the SOII is partially due to the exclusion of injuries and fatalities that involve self-employed agricultural workers, family members working on agricultural operations, and agricultural operations with fewer than 11 employees [13,70]. While AgInjuryNews.org is the largest dataset of its kind, the system is dependent on publicly available U.S. and Canadian media reports, which limits the dataset to only the cases that are reported upon. [15]. Additionally, a dataset that is built on media coverage is subject to selection bias; this limits the generalizability of findings associated with the data. This could also influence the concentration of cases in certain geographical regions and months. The findings should thus be interpreted as describing media-reported incidents only.

## 5. Conclusions

This study presents an assessment of heat-related agricultural illnesses and injuries reported by media outlets in the U.S. based on news reports listed in the AgInjuryNews.org system from 2016 to 2024. The findings suggest that publicly available news reports provide a limited and selective view of heat-related agricultural illnesses and that prevention messaging is often less prominent than investigative framing. News-media-based datasets can therefore be useful for gauging public portrayal of these events. However, these datasets should not be interpreted as representing the burden or distribution of agricultural heat-related illnesses.

The incidence of heat-related illnesses in agriculture is expected to increase as environmental temperatures and humidity continue to rise. Agricultural workers are a high-risk group for heat-related illnesses. To address these risks, OSHA published an Advance Notice of Proposed Rulemaking in 2021 and is reportedly working on launching rulemaking processes to address heat-related workplace health risks [18]. However, these efforts will not extend to smaller agricultural operations that have fewer than 11 employees, with some slight variability between states [71]. The exact number of agricultural operations that will be excluded is unknown; U.S. Economic Research Service data shows that in 2021, about 89% of all farms were small family farms [72]. Thus, the vast majority of agricultural operations could be outside the oversight of the proposed OSHA regulations. Targeted education campaigns on preventing heat-related illnesses by the NIOSH Agricultural Centers, Agricultural Extension Programs, advocacy groups, and other non-profit organizations can be valuable tools to reach smaller agricultural operations. Further research is required to gauge the best method to effectively promote the use of safety strategies that will reduce the risk of heat-related illnesses in agricultural communities. There is a cost associated with such efforts and increased funding should be allocated to address heat-related illnesses.

## Figures and Tables

**Table 1:** The 2016–2024 Agricultural Heat-Related Illnesses from the AgInjuryNews.org dataset.

U.S. State	Year	Month	Victim Age	Fatal/Non-Fatal	Task/Context	Environmental Factors	Occupational Factors	Personal/Health Factors
Florida	2017	May	50	Fatal	Harvesting tomatoes	High temperature	Long fieldwork, inadequate emergency response	Heat exhaustion symptoms
Georgia	2018	June	24	Fatal	Harvesting tomatoes	97 °F (36.1 °C)	Intense exertion	Cardiac arrest from overexertion
Nebraska	2018	July	52	Fatal	Detasseling corn	94 °F (34.4 °C), triple-digit heat index	Outdoor work, inadequate protection	Disorientation
California	2019	July	56	Fatal	Installing irrigation tubing	High ambient temperature	Outdoor work	Collapsed from heat exposure
Missouri	2020	June	26	Fatal	Field labor	Warm, humid day	None noted	Working alone; likely heat exhaustion
Oregon	2021	June	Unk	Fatal	Moving irrigation lines	104 °F (40 °C)	Lack of specific regulations; limited shade/rest	Working alone
Washington	2021	July	69	Fatal	Operating tractor	Low 90 °F (32.2 °C)	Long outdoor shift	Working alone; pre-existing cardiac disease aggravated by heat
Florida	2022	April	35	Fatal	Harvesting strawberries	89 °F (31.7 °C)	Lack of work-rest schedule and training	Disorientation, heat illness
Florida	2023	January	28	Fatal	Weeding/staking bell peppers	78–88 °F (25.6–31.1 °C), heat index 89 °F (31.7 °C)	Long hours, insufficient protection	Fatigue, weakness, disorientation
Minnesota	2023	June	Unknown	Non-fatal	Grain bin rescue (first responder)	Enclosed, hot environment	Physical exertion	—
Minnesota	2023	June	Unknown	Non-fatal	Grain bin rescue (firefighters)	High heat, enclosed space	Prolonged rescue operation	—
Florida	2023	July	30	Fatal	Fieldwork during historic heatwave	Extreme regional heat	Employer neglect, limited protections	Working alone; Reported illness prior to incident
Mississippi	2024	June	74	Fatal	Operating tractor	84 °F (28.9 °C)	None noted	Possible underlying health issues
Arizona	2024	July	26	Fatal	Fieldwork during intense heatwave	116 °F (46.7 °C)	Extreme heat exposure	Collapsed twice; heat stroke

**Table 2:** Article-level mentions of prevention strategies, laws/regulations, and OSHA in the 19 news articles (each article can contain more than one type of content mentioned; hence, the sum is greater than the number of articles).

Content Mentioned in Article	Number of Articles, *n* (%)
Prevention strategies	8 (42%)
Laws or regulations	11 (58%)
State or Federal OSHA	10 (53%)

## Data Availability

Restrictions apply to the availability of these data. Data was obtained from AgInjuryNews.org and is available at https://www.aginjurynews.org/ (accessed on 12 April 2026) with the permission of AgInjuryNews.org.

## Abbreviations

The following abbreviations are used in this manuscript:

U.S.	United States (of America)
OSHA	Occupational Safety and Health Administration
PPE	Personal Protective Equipment
WPS	Worker Protection Standard
AI	Artificial Intelligence
SOII	Survey of Occupational Injuries and Illnesses (by the Bureau of Labor Statistics)
NIOSH	National Institute for Occupational Safety and Health

## Appendix A

**Table A1:** Overview of Heat-Related Illnesses.

Condition	Pathophysiology	Clinical Manifestations
**Heat Stroke** ***Non-exertional heat stroke:*** Heat stroke occurs without engaging in intense physical activity due to physiologic predisposition, anatomic predisposition, medical conditions that impair thermoregulation, high environmental temperatures, inadequate hydration, or inadequate cooling [73]. ***Exertional heat stroke:*** Heat stroke that occurs due to increased physical activity during periods of high ambient temperature and humidity in otherwise healthy individuals [74,75].	When the body is unable to adequately dissipate a significant environmental heat load, this results in elevated core body temperatures. Typically, when the core body temperature exceeds 105 °F (40.5 °C), it can cause central nervous system dysfunction, which is classified as heat stroke [73,76]. Heat stroke can be fatal, and mortality rates of patients presenting to the hospital with the condition range from 21% to 63% [73,76]. Factors such as the degree of core body temperature elevation, the number of organ systems affected, and how quickly cooling mechanisms were initiated are associated with the differences in mortality rate [73,76].	Elevated core body temperature, elevated heart rate, elevated respiratory rate, widened pulse pressure, decreased blood pressure, headache, weakness, lethargy, nausea, dizziness, dilation of blood vessels supplying the skin, fluid buildup in the lungs, problems with blood clotting, altered mental status, slurred speech, irritability, agitation, loss of muscle coordination, loss of muscle control, delirium, seizures, and coma [73,74,76]. Heat stroke can lead to a wide array of long- and short-term complications, including respiratory dysfunction, irregular heartbeat, cardiac dysfunction, hypotension, seizures, cerebral edema, neurologic injury, acute kidney injury, liver injury, disseminated intravascular coagulation, and rhabdomyolysis [73,74,76].
**Rhabdomyolysis**	Rhabdomyolysis is a syndrome marked by muscle cell breakdown that leads to the release of intracellular muscle components into the bloodstream [75]. Cell death can be due to direct damage to the plasma membrane or due to energy depletion [75]. While NIOSH classifies rhabdomyolysis as a heat-related illness, it is important to note that the muscle cell breakdown in rhabdomyolysis can be triggered by a variety of events, including trauma, drug use, toxin exposure, infections, electrolyte disorders, metabolic disorders, myopathies, and hyperthermia [75]. Rhabdomyolysis that is caused by severe heat and humidity is classified as nontraumatic exertional rhabdomyolysis, which is a complication associated with exertional heat stroke [75].	Muscle pain, weakness, dark urine, stiffness, cramping, muscle swelling, swelling in lower legs or hands, skin discoloration, blisters, a general feeling of discomfort, illness, or lack of well-being, fever, elevated heart rate, nausea, vomiting, abdominal pain, irregular heartbeat, cardiac arrest, acute kidney injury, liver injury, and disseminated intravascular coagulation in severe cases [75].
**Heat Exhaustion**	Heat exhaustion occurs when the body is unable to maintain adequate cardiac output due to intense physical activity and high environmental temperatures [73]. It is important to note that for the heat-related illness to be classified as heat exhaustion, there must be no significant central nervous system dysfunction. As mentioned earlier, when central nervous system dysfunction is also present, the illness is classified as a heat stroke.	Elevated heart rate, decreased blood pressure, extreme weakness, dehydration, electrolyte losses, loss of muscle coordination, loss of muscle control, fainting, light-headedness, profuse sweating, “prickly heat” sensations, headache, abdominal cramps, nausea, vomiting, diarrhea, and persistent muscle cramps [73].
**Heat Syncope**	During times of strenuous physical activity in hot ambient temperatures, the increased blood supply to the skin and skeletal muscles can significantly decrease the supply of blood to the brain. When this is coupled with dehydration, it can result in orthostatic dizziness (dizziness when standing up) and fainting [73]. This transient loss or near loss of consciousness due to reduced blood flow to the brain is referred to as heat syncope [73]. It is important to note that exertional heat syncope is not directly caused by heat exposure but arises due to the impact of physical exertion in hot temperatures on the brain’s blood supply. The transient nature of central nervous system involvement and no significant elevation of core body temperature differentiate heat syncope from heat stroke. Heat syncope can also occur in individuals who are not participating in strenuous physical activity. In these cases, the syncopal episode is due to the indirect effects of heat that increase blood supply to the skin and the increased blood pressure required to support an upright posture [73]. Non-exertional heat syncope is often associated with prolonged periods of standing with minimal movement and prolonged periods of sitting with sudden standing [73].	Light-headedness, tunnel vision, pale and sweaty skin, and decreased pulse rate [73]. In most cases, there is rapid recovery with appropriate interventions [73].
**Heat Cramps**	Heat cramps can be described as painful muscle spasms typically affecting the arms, legs, or abdominal muscles [73,74]. Like in heat syncope, heat is not the direct cause for the cramping seen in heat cramps, and, for this reason, the condition is clinically referred to as exercise-associated muscle cramps. The etiology of heat cramps is not fully understood. It is suspected that dehydration, low sodium, low potassium, and fatigue are contributing factors.	Intense muscle pain, muscle spasms, and muscle contractions [73,74].
**Heat Rash**	The clinical condition called eccrine miliaria is commonly referred to as heat rash, sweat rash, or prickly heat [77]. This condition is characterized by the occlusion or inflammation of eccrine sweat ducts, which leads to the build-up of sweat-filled vesicles under the skin that present as a rash [77]. Eccrine miliaria can have several causes, including medication use, cutaneous diseases, disorders that have increased salt in the sweat, ultraviolet radiation, ionizing radiation, bacteria, skin maceration, and genetic disorders [77]. Strenuous physical activity in hot and humid conditions increases the risk of developing eccrine miliaria [77]. Another risk factor pertinent to outdoor workers is the use of non-porous clothing [77]. Based on the depth of sweat duct obstruction, eccrine miliaria is further classified into three main sub-types; this paper will not go into the specifics of the sub-types [77].	Clear superficial vesicles resembling water droplets, erythematous papules, erythematous vesicles, and large, firm flesh-colored papules [77].

## Appendix B

**Table A2:** Narrative Summary of Agricultural Heat-Related Illnesses in AgInjuryNews.org, 2016–2024.

Incident Date	U.S. State	Summary
16 May 2017	Florida	A 50-year-old male farmworker was fatally injured after working in a tomato field. The victim had complained of heat exhaustion during his bus ride back home from the field. He had received water and ice before departing the field, but he asked for help during the journey. His co-worker informed the contractor of the situation and was instructed to let the victim rest and call paramedics once they reached the destination if there was no improvement. When they arrived at the destination, the victim was found unresponsive and not breathing. At the time that the article was published, the County Sheriff’s Office and the Occupational Safety and Health Administration (OSHA) were investigating the incident.
21 June 2018	Georgia	A 24-year-old male farmworker was fatally injured due to a cardiac arrest that was triggered by exposure to extreme heat. He was picking tomatoes in a field when the temperature outside was 97 °F (36.1 °C). The County Coroner stated that the death resulted from over-exertion and heat exhaustion. An Occupational Safety and Health Administration report stated that “The employee was overexerted and began to show symptoms of heat exhaustion. The employee passed out and was taken to a nearby medical facility where he died.” At the time that the report was published, the Occupational Safety and Health Administration was investigating the incident.
13 July 2018	Nebraska	A 52-year-old male migrant farmworker was fatally injured while detasseling corn in a field during high heat. He was disoriented by the high temperature and separated from other workers before experiencing a heat stroke that was fatal. The autopsy report cited heat stroke as the cause of death. The temperature had reached 94 °F (34.4 °C) on the day of the incident. The heat index was in triple digits. At the time when the article was published, the Occupational Safety and Health Administration had proposed penalties to the company that employed the victim for failing to protect employees from excessive heat.
31 July 2019	California	A 56-year-old male farmworker was fatally injured due to heat exposure. He was installing irrigation tubing in an almond orchard when he felt ill, collapsed, and was transported to the hospital, where he later died. The coroner’s report cited complications of heat exposure as the cause of death. At the time of the report, the California Occupational Safety and Health Administration (OSHA) was investigating the incident.
7 June 2020	Missouri	A 26-year-old male farmworker was found fatally injured in a farm pond. The article states that it is believed that he suffered from heat exhaustion and entered the pond to cool off.
26 June 2021	Oregon	A male farmworker was fatally injured due to heat-related causes while working on moving irrigation lines. He was found unresponsive in the field at the end of his shift by his co-workers. The temperatures that day had reached 104 °F (40 °C). Emergency responders attempted CPR but were unable to revive the victim. At the time of the report, the Oregon Occupational Safety and Health Administration (OSHA) was investigating the incident. The article also stated that current regulations require employers to provide water, rest, shade, and training to prevent heat-related illnesses, but the specifics are largely left to employer discretion. The incident and ongoing calls from advocacy groups to implement stricter specific regulations for workplace heat protection in Oregon prompted the state to draft new rules to safeguard outdoor workers from extreme heat.
29 July 2021	Washington	A 69-year-old male farmworker was fatally injured due to atherosclerotic disease with environmental heat as a contributing factor. The determination for cause of death was made by the County Coroner’s Office. He was found slumped over on a tractor in a field by a coworker. The temperatures at the time were in the low 90 °F (32.2 °C). He was pronounced dead at the scene by emergency medical personnel. The coroner noted that heat can place significant stress on individuals with underlying cardiac conditions. The Washington State Department of Labor and Industries released emergency rules requiring employers to provide shade and rest periods for outdoor workers when temperatures reach 100 °F (37.8 °C).
5 April 2022	Florida	A 35-year-old male farmworker was fatally injured due to heat-related illness while harvesting strawberries on a farm. The temperatures outside had reached 89 °F (31.7 °C) on the day of the event. The worker had become disoriented and later unresponsive before he was pronounced dead. The U.S. Department of Labor’s Occupational Safety and Health Administration conducted an investigation into the incident and confirmed that the fatality was caused by heat-related illness. The department also cited the employer for violations such as failing to provide adequate heat illness prevention training and not implementing a work–rest schedule.
1 January 2023	Florida	A 28-year-old male farmworker was fatally injured due to environmental heat exposure after five hours of field tasks (pulling weeds and installing wooden stakes to support bell peppers). The temperature outside ranged from 78 °F (25.6 °C) to 88 °F (31.1 °C), with the heat index reaching a peak of 89 °F (31.7 °C). The worker was observed to be exhibiting signs of heat exhaustion, including fatigue, weakness, and disorientation. He was also observed to be struggling to keep pace with his more experienced co-workers. He had complained of fatigue and leg pain before he was found unresponsive in a drainage ditch by his co-workers. He was pronounced dead by sundown. The U.S. Department of Labor’s Occupational Safety and Health Administration conducted an investigation into the incident and intends to fine the farm labor contractor $15,625 for failing to provide the employee with proper protection.
20 June 2023	Minnesota	A first responder was non-fatally injured due to a heat-related injury during a grain bin rescue. The rescue did not involve a fire; the first responders were rescuing a 27-year-old male who was engulfed by grain.
28 June 2023	Minnesota	Three firefighters were non-fatally injured due to heat exhaustion during a grain bin rescue at a farm. The rescue did not involve a fire; the firefighters were rescuing three individuals who were engulfed by grain.
19 July 2023	Florida	A 30-year-old male farmworker was fatally injured after collapsing while working outside in the heat. He was found unresponsive before being pronounced dead. The victim had stated that he felt sick during a recent shift, which also involved working outside in the heat. The report indicates that it was during a historic heatwave and that this was one of at least two farmworkers who were suspected to be fatally injured from heat exposure in the weeks surrounding the incident. The official cause of death was not released at the time of the article. The article also stated that workers who were interviewed stated that “most bosses aren’t considerate of what workers are going through in this heat”. Protests prompted Miami–Dade County Commissioners to pass a preliminary measure to establish heat safety standards for outdoor workers and also created a new county office to enforce the protections.
27 June 2024	Mississippi	A 74-year-old male was fatally injured while working outside on a tractor. The county coroner confirmed that no trauma was involved and is investigating whether heat was a contributing factor. The temperature outside was around 84 °F (28.9 °C) and the coroner urged caution due to increasing heat conditions.
20 July 2024	Arizona	A 26-year-old male farmworker was fatally injured due to a heat stroke while working in the fields during an intense heatwave. He collapsed twice and was transported to the hospital by co-workers, where he was pronounced dead. The temperatures that day had reached 116 °F (46.7 °C). The Arizona Division of Occupational Safety and Health was investigating the incident at the time of the article. The report states that he was working in extreme heat conditions and that there are no specific state laws in place to protect farmworkers from such heat-related risks.

## Appendix C

**Table A3:** Evidence-Based Strategies for Preventing Heat-Related Illnesses in Agricultural Work.

Intervention	Description
Hydration Measures	Adequate hydration has been shown to reduce the incidence and severity of heat-related illness [22]. Provide employees and workers with easily accessible water stations with clean water and encourage workers to hydrate regularly, ideally in 15–20 min increments. Placing some water stations near restrooms can also increase the use of these stations by improving efficiency [24].
Shade and Rest	In conjunction with hydration, rest and shade have been shown to be effective in reducing heat-related illnesses [18]. Create rest areas that are shaded, cool, and preferably air-conditioned. These areas will only be effective if workers take frequent scheduled rest breaks during peak heat hours.
Heat Acclimatization Strategies	As discussed earlier in this report, heat acclimatization is an important factor that can reduce heat-related illnesses [32,33,57]. Follow the U.S. Department of Health and Human Services guidelines to facilitate heat acclimatization through formal plans [32]. These guidelines list that work time in hot conditions should be gradually increased over a period of 7–14 days, a minimum of 2 h of heat exposure a day is needed to facilitate acclimatization, the intensity of the work performed must be gradually increased to the tasks that are generally required, staying hydrated and eating regular meals during the acclimatization process helps replenish fluids and electrolytes, and improving physical fitness aids the process [32]. Employees who have already been acclimatized but stop working for a week or more must restart the process [32].
Work Scheduling	Reducing the amount of intense physical activity in hot and humid conditions is a key step to prevent heat-related illnesses [61]. Schedule labor-intensive tasks to cooler parts of the workday when feasible to reduce peak heat exposure. Rotate the tasks performed from low intensity to high intensity to prevent overexertion. Utilize scheduled breaks that are frequent to remind employees to take rest in cool, shaded areas. This can also increase employee water consumption.
Personal Protective Equipment	Provide employees with breathable, lightweight clothing that is light-colored when appropriate to the task being performed. If heavy, non-breathable PPE is being used, schedule frequent breaks where employees remove the equipment and rest in cool, shaded areas. Cooling aids such as cooling vests, misting fans, cooling towels, and other cooling equipment can be used to reduce the risk of heat-related illness [20]. Cooling aids are sometimes not used due to limitations in range of motion [20]. Educating employees on the importance of these cooling aids and the relatively larger loss of productivity if the employee develops a heat-related illness could increase the use of the cooling aids.
Education and Training	As mentioned earlier in this report, knowledge gaps about heat-related illnesses can increase the incidence and worsen the outcome of heat-related illnesses [24,34,35,36]. Use formalized education sessions to train workers on how to identify, prevent, and respond to heat-related illnesses. Early intervention and cooling efforts have been shown to improve the outcome of heat-related illnesses [78]. A relevant resource that can help in this process is the Heat Illness Toolkit created by the Pacific Northwest Agricultural Safety and Health Center [79].
Emergency Response Preparedness	Creating and practicing emergency response plans can reduce the extent of injuries [80,81]. Establish a comprehensive heat illness prevention plan that includes a clear protocol for responding to heat-related illnesses. Once a plan is created, make it readily available to employees and provide routine hands-on training to prepare employees to enact the plan.
Monitoring Environmental Conditions	Monitoring environmental conditions and using the information to reduce exposure to unsafe ambient temperatures and humidity can prevent heat-related illnesses [82]. Monitoring environmental conditions involves more than assessing the temperature. Air temperature, relative humidity, radiant heat, and air movement contribute to heat-related illness in workers [82]. To account for all of these factors, the Occupational Safety and Health Administration recommends using a wet bulb globe temperature monitor [82].
Buddy System	The time taken to begin first aid for heat-related illnesses can significantly impact the outcome [78]. Time to discovery can especially be an issue on farms spread across vast distances or difficult terrain. Establish a system where employees are not performing tasks alone. Have other employees present and ensure that all employees know how to recognize and respond to heat-related illnesses.
Special Consideration Based on Age and Chronic Conditions	As mentioned earlier, people over the age of 65, young children, and people with chronic medical conditions are at an increased risk of developing heat-related illnesses [26]. Monitor these individuals more closely for the onset of symptoms. Recommendations for these individuals include more frequent breaks, limited intensity of work being performed, and utilization of additional cooling measures.
Special Consideration for Migrant and Seasonal Farmworker Populations	Migrant and seasonal farmworkers face a disproportionately high risk of developing heat-related illnesses due to multiple structural factors [18,44,45,46]. Comprehensively addressing farmworker health disparities related to heat illnesses is beyond the scope of this manuscript. However, farm-level interventions, in addition to the recommendations listed above, include the following: ensure that educational materials and protocols developed are available in the worker’s language; deliver educational materials and protocols in a verbal format for workers that cannot comprehend written language; stop utilizing piece-rate payment methods; provide quality housing that is safe, has adequate ventilation and temperature control, and is not overcrowded; identify local health-care facilities that do not verify immigration or citizenship status and share this information with workers; and implement measures to prevent fear of retaliation if the worker reports heat-related illnesses [18,24,83,84,85].
